# A Transparent Polymer-Composite Film for Window Energy Conservation

**DOI:** 10.1007/s40820-025-01668-6

**Published:** 2025-02-17

**Authors:** Xianhu Liu, Haoyu Zhang, Yamin Pan, Jun Ma, Chuntai Liu, Changyu Shen

**Affiliations:** 1 College of Materials Science and Engineering, State Key Laboratory of Structural Analysis, Optimization and CAE Software for Industrial Equipment, National Engineering Research Center for Advanced Polymer Processing Technology, Zhengzhou University, Zhengzhou, 450002 People’s Republic of China; 2 UniSA STEM and Future Industries Institute, University of South Australia, Adelaide, SA 5095 Australia

**Keywords:** Energy conservation, Polymer, Transparent films, Composite, Radiative cooling

## Abstract

As living standards improve, the energy consumption for regulating indoor temperature keeps increasing. Windows, in particular, enhance indoor brightness but also lead to increased energy loss, especially in sunny weather. Developing a product that can maintain indoor brightness while reducing energy consumption is a challenge. We developed a facile, spectrally selective transparent ultrahigh-molecular-weight polyethylene composite film to address this trade-off. It is based on a blend of antimony-doped tin oxide and then spin-coated hydrophobic fumed silica, achieving a high visible light transmittance (> 70%) and high shielding rates for ultraviolet (> 90%) and near-infrared (> 70%). When applied to the acrylic window of containers and placed outside, this film can cause a 10 °C temperature drop compared to a pure polymer film. Moreover, in building energy simulations, the annual energy savings could be between 14.1% ~ 31.9% per year. The development of energy-efficient and eco-friendly transparent films is crucial for reducing energy consumption and promoting sustainability in the window environment.

**Supplementary Information:**

The online version contains supplementary material available at 10.1007/s40820-025-01668-6.

## Highlights

- The resultant film offers high visible light transmittance (>70%) while effectively blocking UV (>90%) and NIR (>70%) radiation, addressing the balance between natural lighting and solar energy control.
- Incorporating hydrophobic silica, it achieves high emissivity for radiative cooling, reducing indoor temperatures by up to 10 °C and achieving maximum additional cooling energy savings of 261 MJ m^−2^ per year.
- Its superhydrophobic surface ensures excellent self-cleaning properties and durability, making it highly suitable for long-term outdoor applications.

**Supplementary Information:**

The online version contains supplementary material available at 10.1007/s40820-025-01668-6.

## Graphical abstract

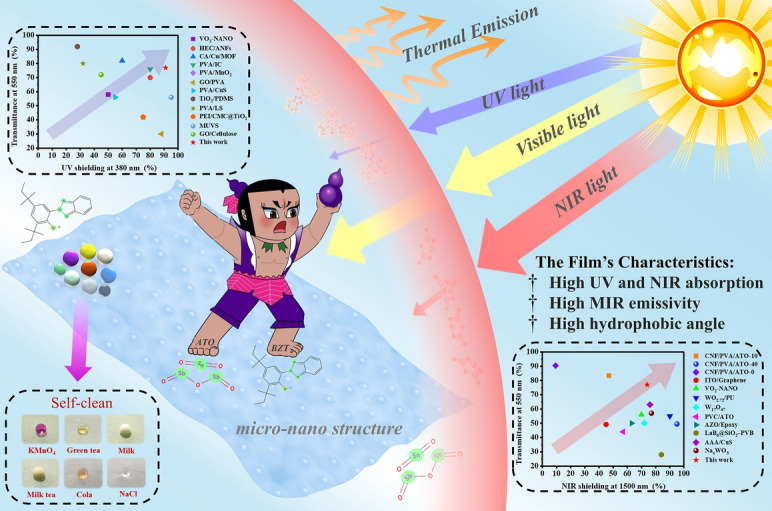

## Introduction

Energy consumption from buildings, including indoor cooling and heating, is rapidly increasing and now accounts for 40% of total energy use in developed countries [1–3]. Windows amplify the energy demands for cooling and heating interiors, and as the least energy-efficient component of a building, can account for as much as 60% of the building’s energy consumption [4–6]. Therefore, the development of smart window technology is a paramount importance for enhancing the energy efficiency of buildings. Such technology should not only effectively reduce heat transfer through the windows, but also preserve indoor adequate natural light and visual comfort.

Radiative cooling, where thermal energy is emitted into outer space through black body radiation [7, 8], offers a viable strategy to reduce indoor cooling energy consumption. High emissivity in the atmospheric window (wavelength 8 < λ < 13 μm) lowers the internal temperature of an object, thereby decreasing energy consumption [9]. Various materials have been proposed for radiative cooling, including porous materials [10–12], multilayer materials [13–15], photonic crystals [16], and biomimetic materials [17, 18]. Additionally, practical applications of radiative cooling, such as self-regulation in response to external temperature changes [19] and the development of fabrics for passive radiative cooling of the human body, have also been explored [20, 21]. However, traditional materials used for radiative cooling all have low transmittance in the solar spectrum (0.3 < λ < 2.5 μm), and opaque materials (white or silver) with high reflectivity are usually adopted to maximize the radiative cooling capacity [22–27]. These material properties conflict with the need for high transmittance in the visible range (0.38 < λ < 0.76 μm) for indoor lighting, so indoor cooling via radiative cooling remains challenging. The ideal cooling material for window applications should be highly transparent in the visible spectrum, which constitutes about 43% of total solar energy. It should also provide strong shielding in the ultraviolet (UV) and near-infrared (NIR) spectra, which together account for approximately 57% of total solar energy [28, 29].

Recently, visible-light transparent cooling materials composed of nanofunctional fillers such as silicon dioxide (SiO_2_) microspheres and zinc oxide (ZnO) nanoparticles have gradually attracted attention [30–33]. Studies have shown that the NIR transmittance of SiO_2_ microspheres can be effectively reduced by controlling their size [6, 34]. However, composite films doped with SiO_2_ or ZnO exhibit poor shielding effectiveness in the NIR range, leading to insufficient shielding in the UV-NIR spectrum. Thus, higher filler concentrations are often required to meet the demands of practical applications. Furthermore, materials highly transparent in the visible region such as indium tin oxide (ITO) [35] and antimony tin oxide (ATO) [36, 37] have low transmittance in the NIR region because of their free carrier absorption in the conduction band. Compared to SiO_2_ and ZnO, ITO and ATO have superior shielding efficiency in the NIR spectrum, allowing for effective blocking at lower loading levels, and ATO has better economic benefits relative to ITO, which better meets practical application requirements. Currently, research on the application of ATO composite materials for transparent smart windows is limited, leaving this area relatively underexplored.

High-emissivity materials play a pivotal role in radiative cooling, enabling passive heat dissipation by efficiently radiating thermal energy into outer space through the mid-infrared atmospheric windows (8–13 and 16–24 µm) [38–42]. This mechanism operates without additional energy input, offering an eco-friendly and sustainable solution for building energy efficiency. To enhance sample emissivity, hydrophobic fumed silica (Hf-SiO_2_) is a cost-effective material with exceptional emissivity properties. The nanoscale size of Hf-SiO_2_ creates a rough, highly scattering surface, significantly boosting radiation emission in the mid-infrared range. Additionally, Hf-SiO_2_ exhibits excellent thermal stability, ensuring long-term reliability for large-scale applications. The spin-coating process is straightforward and highly controllable, enabling uniform thin-film fabrication, improving efficiency, and maintaining consistent material performance. This approach, integrating material advantages and process simplicity, paves the way for the development of high-emissivity radiative cooling materials and accelerates their practical implementation in energy conservation and environmental sustainability.

Herein, we developed a transparent cooling film for application to the surfaces of windows (building or car) with excellent UV and NIR dual shielding performance based on the functional group matching principle and material properties. The transparent composite film was prepared by mixing ATO and 2-(2H-benzotriazol-2-yl)-4, 6-ditertpentylphenol (BZT) with ultrahigh-molecular-weight polyethylene (UHMWPE), followed by hot pressing the resulting composite film with sandpaper and spin-coating Hf-SiO_2_ onto the surface. The first process was designed to enable the resulting film to transmit visible light while blocking UV and NIR light. The second process aimed to impart high emissivity to the film in the atmospheric window, allowing it to radiate heat to the surrounding environment through localized surface plasmon resonance [43] and chemical bond vibration frequencies [44]. We then used this film in outdoor tests as a coating film on the window of a small container; this effectively reduced the temperature inside the container by 10 °C in the summer relative to the temperature of an analogous container covered with a pure UHMWPE film. Additionally, the prepared film exhibited superhydrophobicity imparted by the sandpaper-assisted molding process, resulting in excellent resistance to the buildup of pollutant particles. This transparent cooling film is expected to be suitable for broad applications to energy saving for windows and other glass systems.

## Experimental Section

### Materials

UHMWPE powder with Mn of (2.0–3.0) × 10^6^ g mol^−1^ was supplied by Beijing Eastern Petrochemical Co., Ltd. BZT was purchased from Shanghai Aladdin Bio-Chem Technology Co., Ltd., China. ATO was purchased from MCC South Cold Rolling New Material Technology Co., Ltd., China. Hf-SiO_2_ (110 m^2^ g^−1^ specific surface area, 16 nm diameter, and 0.6–1.2 wt% carbon content) was purchased from Evonik Industries Co., Ltd., Germany. Ethanol (C_2_H_5_OH) was obtained from Tianjin Damao Chemical Reagent Co., Ltd. Xylene (analytical reagent, 99%) was purchased from Damao Chemical Reagent Co., Ltd. Antioxidant Irganox 1010 was purchased from Dinghai Plastic Chemical Co., Ltd. All reagents were used directly without further purification.

### Preparation of Smooth UHMWPE Composite Films

The composite films were prepared by solution blending. ATO (10 wt% to UHMWPE) and BZT (2 wt% to UHMWPE) were ultrasonic in 80 mL xylene for 2 h. Then, 300 mg of UHMWPE powder and Irganox 1010 (0.05 wt% to UHMWPE) were added into the dispersion by ultrasound for 30 min, and the mixture was then stirred in an oil bath at 135 °C for 2 h until the solution was colorless and translucent. Then, the solution pours into the Teflon mold and evaporates the solvent at room temperature to procure the film. The film was then sandwiched in the following order within a vacuum film press: 304 stainless steel plate—polyimide film—ATO composite film—polyimide film—304 stainless steel plate. The film press was set to a temperature of 150 °C, a pressure of 39.8 kN, with a heating time of 10 min, and a holding time of 30 min, this process yielded a smooth ATO composite film.

### Preparation of Micro-Structured UHMWPE Composite Films

The above-prepared film was placed in the vacuum film pressing machine (120 °C, 39.8 KN) in the order of 304 stainless steel—polyimide—UHMWPE composite film—sandpaper—polyimide—304 stainless steel, heated for 10 min, and kept pressure for 10 min.

### Preparation of Micro-Nanostructured UHMWPE Composite Films

The Hf-SiO_2_ was dispersed in ethanol by ultrasonic to form a dispersion solution with a concentration of 100 mg mL^−1^. The film is placed on a PC substrate and treated with plasma radiation for 90 s. After that, Hf-SiO_2_/ethanol was spin-coated onto UHMWPE composite film at 500 rpm for 10 s, followed by 800 rpm for 10 s to form a micro-nanostructure surface, which was dried at room temperature to form the film.

### Materials Characterizations and Measurements

The surface morphology of the composite membrane was characterized using a scanning electron microscope (JSM-7001F). A super depth-of-field microscope (DVM6 A) was utilized to analyze the three-dimensional morphology of the micro- and nanostructures on the membrane's surface. The water contact angle (WCA) of different samples was measured using a contact angle measuring instrument (SL200KS). The haze of the composite membrane was evaluated with a BYK Transmission Haze Shadow Meter (BYK-4775). A UV–Vis-NIR spectrophotometer (Cary 5000) was employed to measure the transmittance and reflectance of the samples across the solar wavelength range (0.25–2.5 µm). The transmittance (T) and reflectance (R) in the mid-infrared range (2.5–25 µm) were characterized using a Fourier transform infrared spectrometer (Nicolet 6700), with emissivity (E) calculated as E (%) = 100%—T (%)—R (%). Solar irradiance was measured using an optical power meter (CEL-FZ-A). The Jinko (JK808) was used to record real-time temperature changes in the outdoor test chamber. EnergyPlus 9.2.0 was employed to simulate building energy consumption across different regions.

## Results and Discussion

### Design and Fabrication of the Micro-Nanostructures and Superhydrophobicity Characterization

**Fig. 1:**
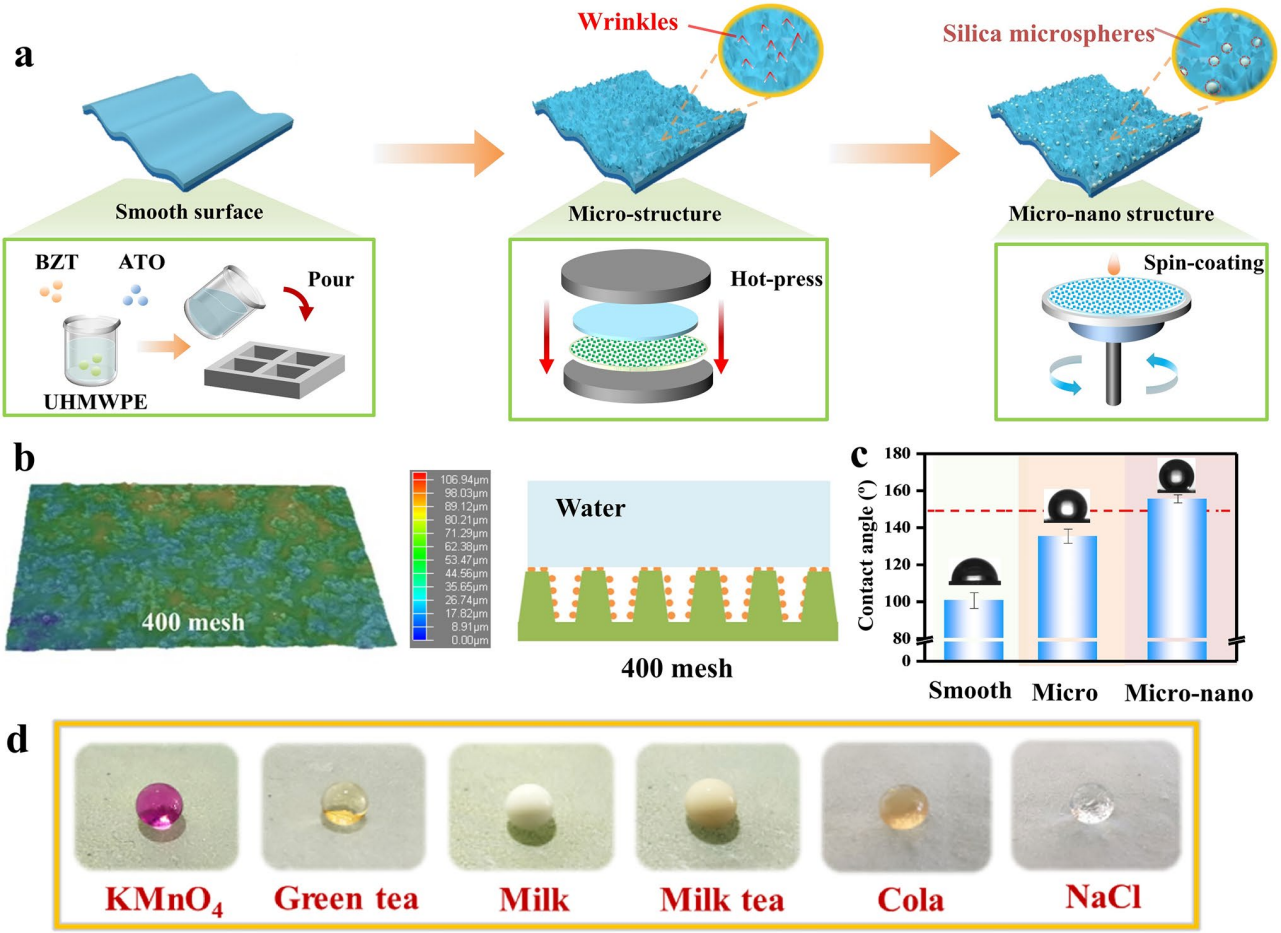
Fabrication and superhydrophobicity of the transparent polymer composite film. **a** Fabrication process of the films. **b** 3D image of the film surface (400-mesh sandpaper) and its diagram of the contact angle of a droplet on the surface. **c** Water contact angles on the films with various surfaces. **d** Photos of common liquid droplets on the film surface

Preparing the films involved three main steps (Fig. 1a): (i) solution blending and molding to form a polymer composite film with a smooth surface; (ii) hot pressing with sandpaper to create a microscale surface texture; (iii) spin coating with hydrophobic fumed silica (Hf-SiO_2_) to achieve micro- and nanoscale structuring. The resulting transparent cooling film was labeled PE-AB-Si, while the film using only ATO as a filler was labeled PE-A.

Herein, to achieve optimal hydrophobicity and solar reflection, sandpapers with varying mesh sizes were used in step (ii) to prepare the films for comparison. The hydrophobicity of these films can be explained in terms of the Cassie–Baxter model [45] that derives from their morphologies (Figs. 1b, S1) [46]. This assumption was further confirmed by measuring the contact angles of the smooth surfaces, microstructure surfaces, and micro-nanostructured surfaces (Figs. 1c, S2). Photothermal experiments confirmed that sandpaper with higher roughness created a larger specific surface area on the film’s surface, enhancing its contact with sunlight (Fig. S3). Consequently, films prepared with 400-mesh sandpaper were chosen for further study.

Considering the harsh outdoor environment, the application of the developed films on the outside of buildings necessitates robust superhydrophobic capabilities. For example, in outdoor applications where dust is unavoidable, self-cleaning behavior helps maintain the transparency of the glazing system. Thus, the self-cleaning capability of the PE-AB-Si film was tested. The dust (KMnO_4_ powder) was easily removed from the film surface without leaving traces by applying water droplets (Fig. S5, Video S1). This demonstrates that PE-AB-Si films can achieve self-cleaning outdoors through natural rainfall. Meanwhile, various common liquids as shown in Fig. 1d (Fig. S5a) were applied to test the hydrophobicity of the film, and the results showed that its surface was sufficient to repel liquids with different natures. Additionally, in the water adhesion tests, the water droplets remained intact and were easily removed by the needle (Video S2). These results indicate low water adhesion on the surface of PE-AB-Si film. Furthermore, the film maintains a contact angle of 150° in both acidic and alkaline conditions (Fig. S4), indicating its ability to retain superhydrophobicity and self-cleaning properties in complex outdoor environments (e.g., outdoor fugitive dust, Video S3).

### Optical Properties of Composite Films

**Fig. 2:**
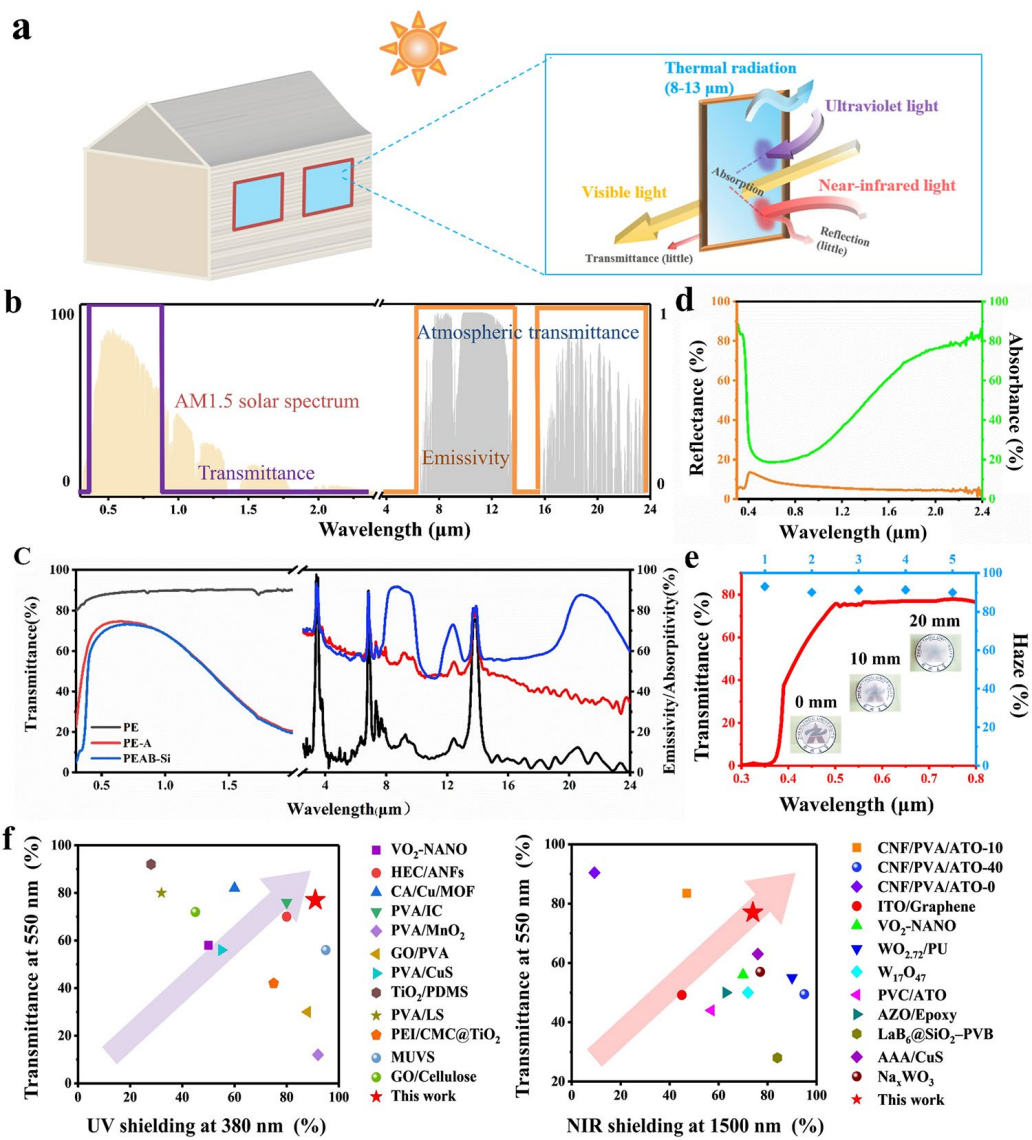
Optical properties of the transparent polymer composite film. **a** Cooling principle of the film. **b** Transmittance and emissivity spectra of an ideal transparent cooling material. The spectral irradiance was determined from the standard direct spectrum (AM1.5d). **c** Transmittance and emissivity spectra of films tested in this study. **d** Reflectance and absorbance spectra of film. **e** Haze of film; the insets show photographs of a film placed at different heights above a background image. **f** Overview of the UV and NIR shielding of various transparent films. Data for comparison were obtained from the literature; see Table S1

According to Kirchhoff's law [47], under thermodynamic equilibrium, the monochromatic radiative emittance and absorptance of different objects at the same wavelength are equal. This value matches the monochromatic radiative emittance of a blackbody at the same wavelength and temperature. Thus, a completely transparent object does not radiate heat. Near-infrared light (NIR, 0.76 < λ < 2.4 μm), which makes up 50% of sunlight, carries significant heat. Ultraviolet light (UV, 0.3 < λ < 0.38 μm), accounting for 7% of sunlight, not only contributes to heat, but also accelerates the deterioration of materials and indoor furnishings, posing potential health risks to occupants. When transparent materials are used in outdoor buildings, the primary cause of increased indoor temperatures is the entry of NIR and UV into the interior. To develop transparent films with optimal cooling effects, it is essential to ensure high transmittance of visible light (0.38 < λ < 0.76 μm), which accounts for 43% of sunlight, while minimizing the transmittance of light with other wavelengths. At the same time, a high emissivity in the atmospheric window (wavelength: 8 < λ < 13 μm) is necessary to ensure that the thermal radiation of the internal space can be effectively emitted (Fig. 2a). Specifically, an ideal transparent cooling material is expected to have high shielding against UV and NIR light, high transmittance of visible light, and high emissivity in the atmospheric window, as shown in Fig. 2b.

The pure PE film showed high transmittance at all wavelengths (Fig. 2c), so when this film is applied to building windows, a large amount of light will enter a room, increasing the indoor temperature. The PE-A film can shield a large amount of NIR light and some UV light; however, it also has low emissivity in the atmospheric window, suggesting that it cannot actively emit the internal thermal radiation to the surrounding environment. In contrast, the film of PE-AB-Si not only has a strong shielding effect for NIR and UV light, but also has a high emissivity in the atmospheric window (84.6%), so it can effectively release internal thermal radiation. Next, the direction of the shielded NIR and UV light was studied. The results show that most of the NIR and UV light was absorbed by the film, and only a small amount was reflected (Fig. 2d). In addition, film exhibited high haze, which helps diffuse the light passing through the film. This film was placed at heights of 0, 10, and 20 mm from a background image, and the image became blurred as the height increased (Fig. 2e). This feature is particularly important in buildings where privacy is needed or in glass conservatories.

Additionally, the UV/NIR shielding performance and visible light transmission performance of the film of PE-AB-Si were compared with those of other transparent films. Details about the properties of these films are provided in Fig. 2f, where the direction of the arrows indicates the trend of overall performance improvement. Typically, there is a trade-off between UV/NIR shielding and visible light transmittance. However, the PE-AB-Si film demonstrated both high UV/NIR shielding efficiency and excellent visible light transmittance compared to other films with similar properties.

### Outdoor Cooling Performance Evaluation of the Composite Films

**Fig. 3:**
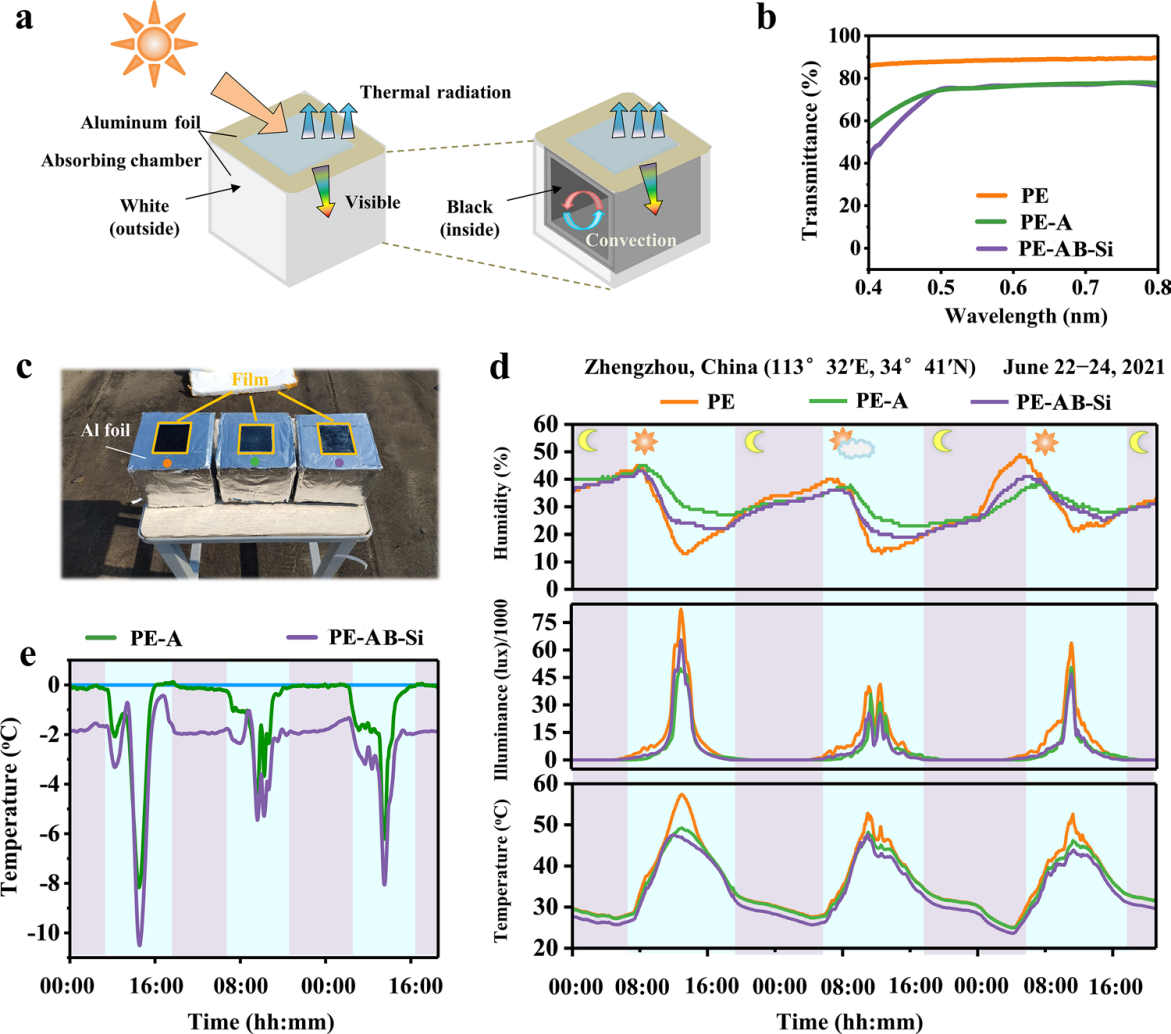
Cooling performance of the transparent polymer composite film. **a** Schematic diagram of the outdoor testing apparatus. **b** Transmittance of the three films tested in visible light. **c** Photograph of the outdoor refrigeration performance measurement system. **d** Realistic outdoor relative humidity, solar illumination, and temperature in Zhengzhou, China. **e** Net cooling provided by the film (obtained by subtracting from the pure PE film data)

To test the cooling abilities of the developed film, we placed the film on transparent acrylic containers and then placed these containers outside, as depicted in Fig. 3a, c. (Specific details about the containers are provided in Note S1). The interior of the containers was heated by sunlight passing through the transparent window. Compared to pure PE windows, PE-AB-Si films effectively block most UV and near-infrared rays while preserving favorable visible light transmittance (> 70%). The film's high emittance within the atmospheric window further facilitated the active release of heat from the cavity. Other test results and the weather conditions are shown in Fig. 3d. The illuminance inside the containers with PE-A and PE-AB-Si film windows decreases slightly, consistent with the results in Fig. 3b, but the overall transmittance was still ideal; this phenomenon occurred under both sunny and cloudy weather conditions. The temperature of the PE-A film-covered container was approximately 8 and 3.5 °C lower than that of the container with PE on sunny and cloudy days, respectively, while the temperature of the PE-AB-Si film-covered container was 10 and 5 °C lower (Fig. 3e). The temperature difference observed in the PE-A-covered container was primarily due to the film's effective UV/NIR absorption, which minimized energy absorption within the cavity. PE-AB-Si film, in addition to absorbing UV/NIR, exhibits exceptional radiative cooling capabilities, enabling internal heat to be emitted to the external environment and further lowering the cavity temperature. Notably, the temperature difference between the PE-A and PE-AB-Si film-covered containers was approximately 2 °C, which is smaller than the forecasted conclusion in our previous study (Fig. 2c). This is primarily because cavity, like real building, possesses a certain level of emissivity.

### Energy Consumption Simulation for Composite Films

**Fig. 4:**
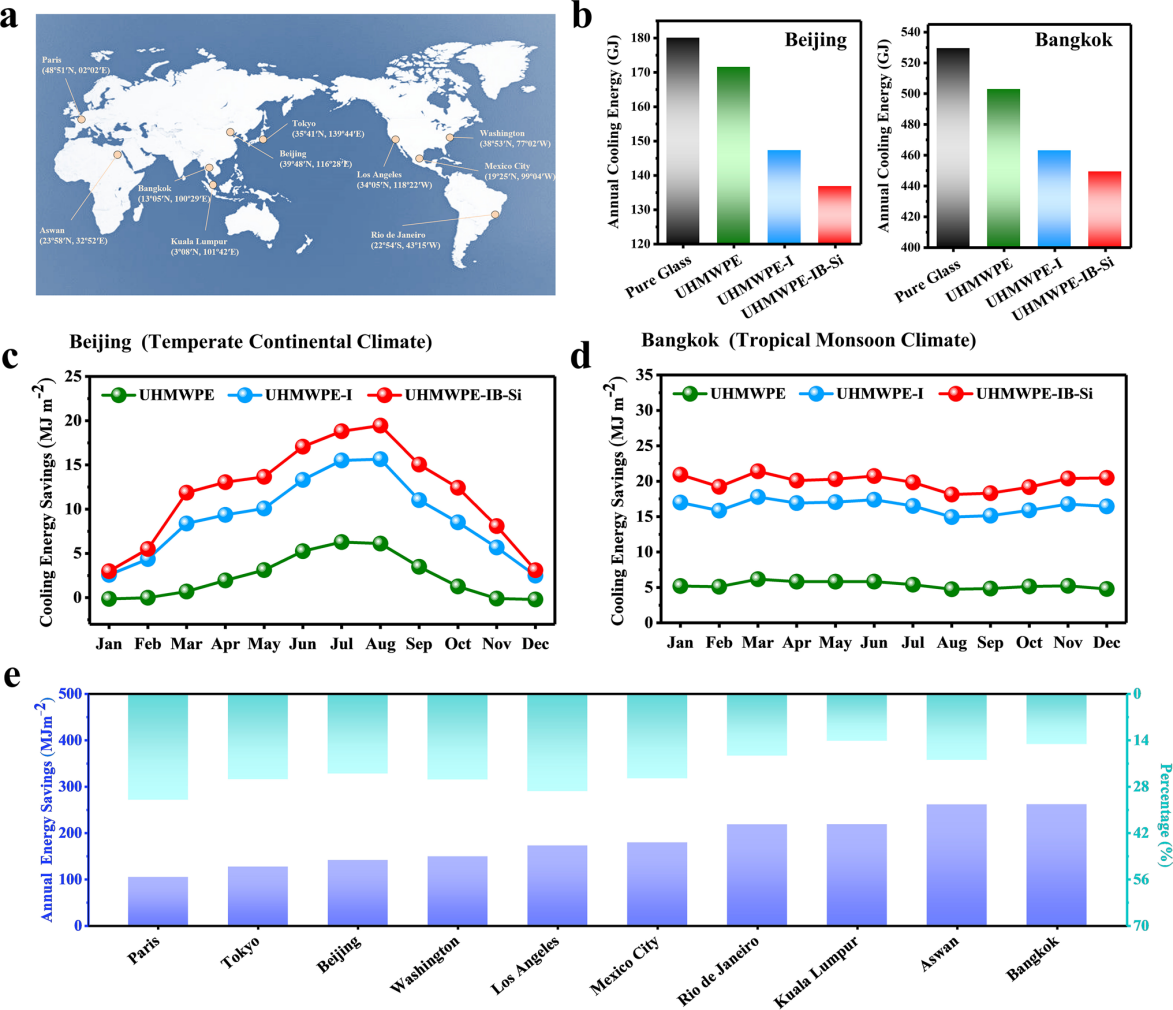
Modeling the cooling energy consumption reducing with the application of the transparent polymer composite film to windows. **a** Geographic location, latitude, and longitude of 10 representative cities. **b** Annual energy consumption for cooling of the model buildings using four types of windows based on the weather data of Beijing and Bangkok. **c**, **d** Cooling energy consumption reducing of building models using three types of film-coated windows relative to that of a building with bare glass windows in **c** Beijing and **d** Bangkok each month. **e** Annual cooling energy consumption reducing and percentage of energy consumption reducing for the model buildings using the PE-AB-Si film based on weather data from 10 cities

To assess the practical application potential, the energy-saving performance of the developed films were simulated using a typical mid-rise apartment model (see Note S2 for details) across 10 cities with diverse climates. Their geographic locations are shown in Fig. 4a, with coordinates and climate details provided in Table S2. The annual cooling energy consumption for Beijing and Bangkok, which have a medium-latitude monsoon climate and a tropical monsoon climate, respectively, is shown in Fig. 4b. The annual energy savings reducing of the pure PE film relative to those of bare glass are only 27.7 and 86.6 MJ m^−2^ for Beijing and Bangkok (Fig. S15), respectively, which is attributed to the low selectivity of the PE film for light transmission and the lack of emissivity in the atmospheric window. In contrast, due to the dual UV and NIR absorption characteristic and high emissivity in the atmospheric window, the annual energy savings reducing of the PE-AB-Si film are 141.1 and 261.6 MJ m^−2^ (Fig. 4c), which are 5 times and 3 times higher than those of the pure PE film, respectively. Because the temperature in tropical areas remains high throughout the year, the monthly cooling energy consumption reducing provided by the film is always maintained at a high level. By contrast, in monsoon climates at medium latitudes, seasonal temperature variations result in cooling energy consumption that peaks in the summer and reaches a trough in the winter. This trend also meets the needs of practical application (Fig. 4c, d). This result indicates that the film of PE-AB-Si has good energy-reducing potential in both temperate and tropical climates.

The simulation results of the other 8 cities show that the annual cooling energy consumption reducing provided by the film are between 104.3 and 261.2 MJ m^−2^ (Fig. S13; Paris, France (104.3 MJ m^−2^); Tokyo, Japan (126.9 MJ m^−2^); Washington, USA (149.1 MJ m^−2^); Los Angeles, USA (172.5 MJ m^−2^); Mexico City, Mexico (179.3 MJ m^−2^); Rio de Janeiro, Brazil (218.2 MJ m^−2^); Kuala Lumpur, Malaysia (218.4 MJ m^−2^); and Aswan, Egypt (261.2 MJ m^−2^)). In addition, the monthly cooling energy consumption reducing show a consistent trend with the temperature changes in these 8 cities (Fig. S14). Notably, the predicted annual cooling savings in cities located in tropical regions are significantly higher than those in subtropical and temperate regions. However, the trend in terms of the percentage reduction in energy consumption is the opposite (Fig. 4e). Cities in temperate and subtropical regions, such as Paris (31.9%) and Los Angeles (29.4%), show a higher percentage reduction compared to cities in tropical regions like Kuala Lumpur (14.1%) and Aswan (19.9%). Compared to the latter cities, the former cities experience shorter periods of direct sunlight, have lower average temperatures, and consume less total annual cooling energy, which results in a more significant percentage reduction in energy consumption. Nevertheless, the annual cooling savings and the energy-saving percentages of the PE-AB-Si film are still much higher than those of the other two PE films (Figs. S15, S16). The above simulation results show that the PE-AB-Si film has good cooling capacity under different climatic conditions, which will help expand its application field and development potential.

## Conclusions

In summary, this work presents a transparent radiative cooling film and demonstrates its cooling abilities in outdoor experiments. The developed film has high transmittance in the visible light region (> 70%), high absorption of the UV (> 90%) and NIR (> 70%) regions, and high emissivity (84.6%) in the atmospheric window, satisfying both transparence and cooling requirements. Compared with a bare sample, the proposed film blocks solar energy in nonvisible regions and effectively lowers the temperature. Our experiments demonstrate that the PE-AB-Si film reduces heat absorption in the system and provides a radiative cooling effect. The temperature of the PE-AB-Si film is approximately 10 °C lower compared to PE film. In addition, the PE-AB-Si film has good hydrophobic and self-cleaning properties due to its surface micro-nanostructure, which suggests its suitability as a protective coating film for window systems. This developed film aligns with the current social trend of promoting a low carbon footprint and energy conservation. It achieves energy savings through low-cost preparation, while seamlessly combining aesthetics and functionality, offering excellent application potential for future window systems.

## Supplementary Information

Below is the link to the electronic supplementary material.

Supplementary file1 (MP4 1214 KB)

Supplementary file2 (MP4 1385 KB)

Supplementary file3 (MP4 2472 KB)

Supplementary file4 (DOCX 7402 KB)
