# Supplementary material for: A Transparent Polymer-Composite Film for Window Energy Conservation
Source: Nanomicro Lett. 2025 Feb 17;17:151. doi: 10.1007/s40820-025-01668-6 (PMC11832844; doi:10.1007/s40820-025-01668-6)
Supplement: Supplementary file 4 — Supplementary file4 (DOCX 7402 KB) [file 40820_2025_1668_MOESM4_ESM.docx]

Supporting Information for

**A Transparent Polymer-Composite Film for Window Energy Conservation**

Xianhu Liu^1,2^, Haoyu Zhang^1^, Yamin Pan^1,^ *, Jun Ma^2^, Chuntai Liu^1^, Changyu Shen^1^

^1^ College of Materials Science and Engineering, State Key Laboratory of Structural Analysis, Optimization and CAE Software for Industrial Equipment, National Engineering Research Center for Advanced Polymer Processing Technology, Zhengzhou University, Zhengzhou 450002, P. R. China

^2^ UniSA STEM and Future Industries Institute, University of South Australia, SA, 5095, Australia

*Corresponding author. E-mail: [yamin.pan@zzu.edu.cn](mailto:yamin.pan@zzu.edu.cn) (Yamin Pan)

**Note S1 Cooling Device Design**

The acrylic cube was wrapped in polypropylene foam and aluminum foil to reduce the effects of heat convection and heat conduction, and the device was placed at a certain height above the ground to reduce the effects of heat radiation near the ground. Sensors were placed inside the three chambers while temperature, humidity, and illumination were measured for three days.

**Note S2 Theoretical simulation**

EnergyPlus (version 9.2.0) software was used to simulate the energy consumption to evaluate the energy-saving effect of UHMWPE composite films. The building model used in the simulation was a typical midrise apartment building, and the coefficient of lighting consumption and internal load were modified based on the U.S. Department of Energy commercial reference building models. The total building area is 3134.61 m^2^, the net conditioned building area is 2823.98 m^2^ and the window opening area is 306.92 m^2^. We used the weather data from the website to calculate the annual energy consumption of the building model in 10 cities around the world1. We neglected the effect of thermal resistance due to the negligible thickness of the composite film and measured UV-VIS-NIR and infrared spectra were used in the simulation.

The calculations of integral solar transmittance T_solar_ (0.3-2.5 μm) and thermal emittance ε (2.5-20 μm) can be found in Eqs. (S2.1) and (S2.2), respectively:

$$\begin{aligned} T_{\mathrm{solar}}=\frac{\int_{0.3}^{2.5} d\lambda\cdot t\left( \lambda\right)\cdot I_{AM.1.5}\left( \lambda\right)}{\int_{0.3}^{2.5} d\lambda\cdot I_{AM.1.5}\left( \lambda\right)}\#\left( S2.1 \right) \end{aligned}$$

$$\begin{aligned} \varepsilon=\frac{\int_{2.5}^{20} d\lambda\cdot\varepsilon\left( \lambda\right)\cdot I_{BB}\left( T,\lambda\right)}{\int_{2.5}^{20} d\lambda\cdot I_{BB}\left( T,\lambda\right)}\#\left( S2.2 \right) \end{aligned}$$

and visible light transmittance T_vis_ (0.4-0.76 μm) can be expressed as Eq. (S2.3):

$$\begin{aligned} T_{\mathrm{vis}}=\frac{\int_{0.4}^{0.76} d\lambda\cdot t\left( \lambda\right)\cdot I_{AM.1.5}\left( \lambda\right)}{\int_{0.4}^{0.76} d\lambda\cdot I_{AM.1.5}\left( \lambda\right)}\#\left( S2.3 \right) \end{aligned}$$

where *I*_AM1.5_(*λ*) represents the solar illumination of the AM 1.5 spectrum and *I*_BB_ (*T*, *λ*) is the spectral radiance of a blackbody at temperature *T. t* (*λ*) and ε (*λ*) are the transmittance and emittance of the film corresponding to the related wavelength range, respectively.

**Supplementary Figures**


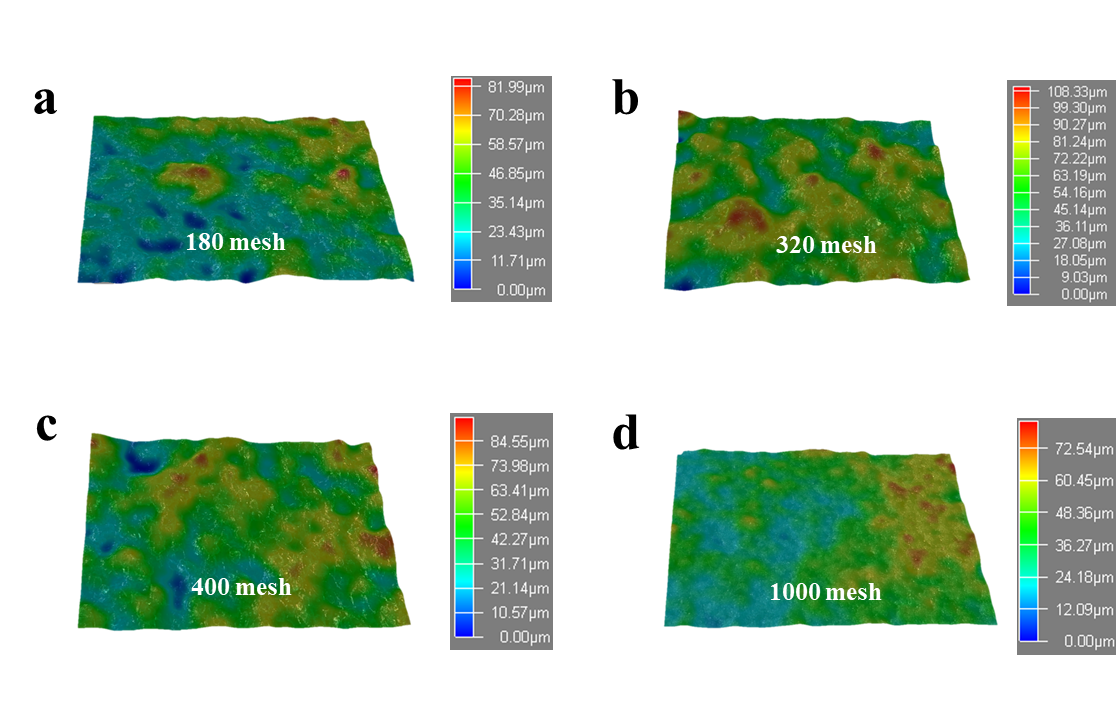


**Fig. S1** 3D views of sandpaper of different meshes (180, 320, 400, and 1000 mesh)


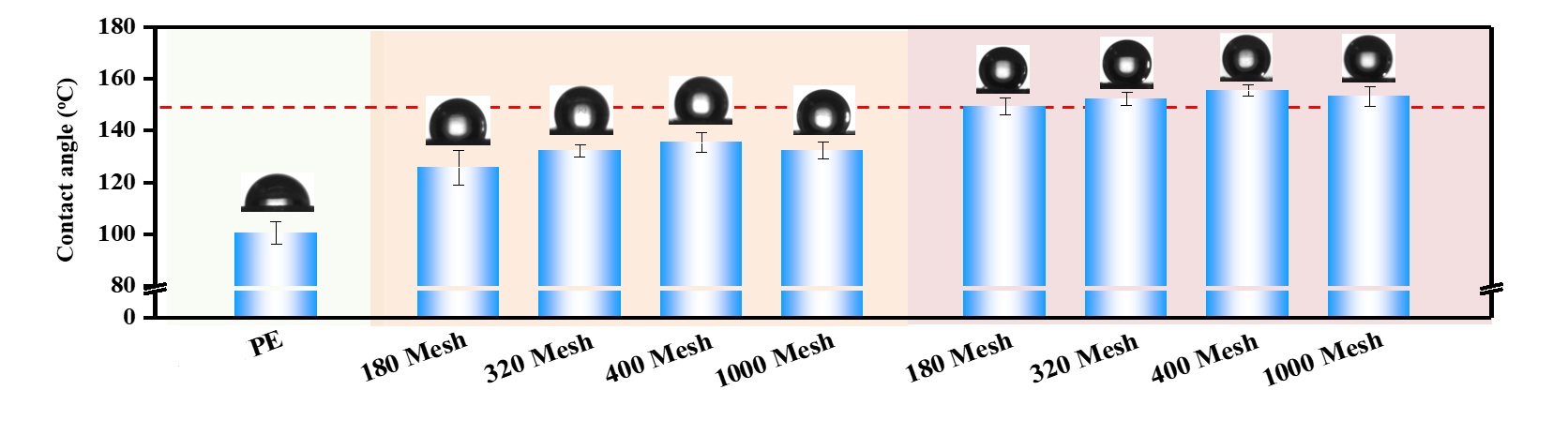


**Fig. S2** Water contact angles on the films with smooth surface and four different mesh surfaces
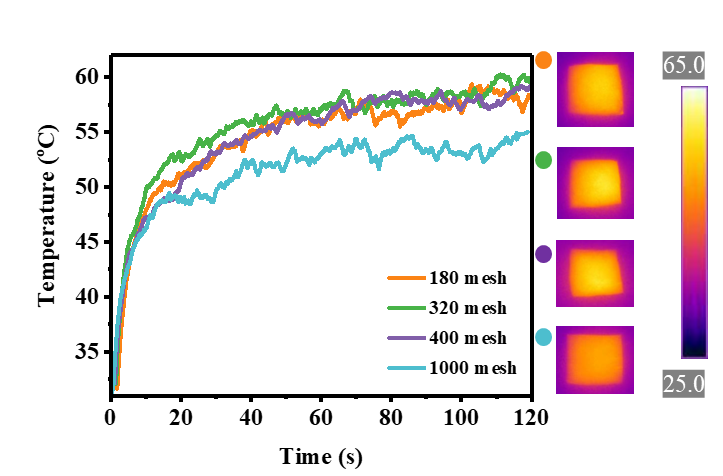


**Fig. S3** Photothermal effects of films with four different mesh surfaces (illustrated with corresponding IR images)


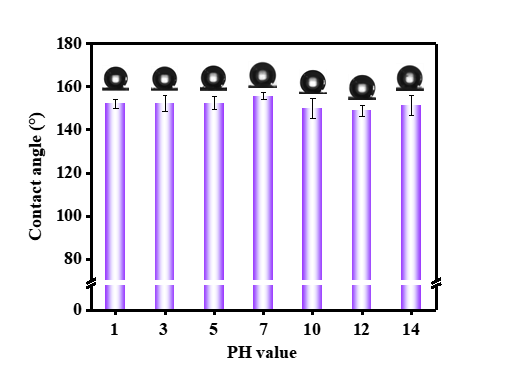


**Fig. S4** Contact angles of aqueous solutions with different pH values on the surface of composite films

**
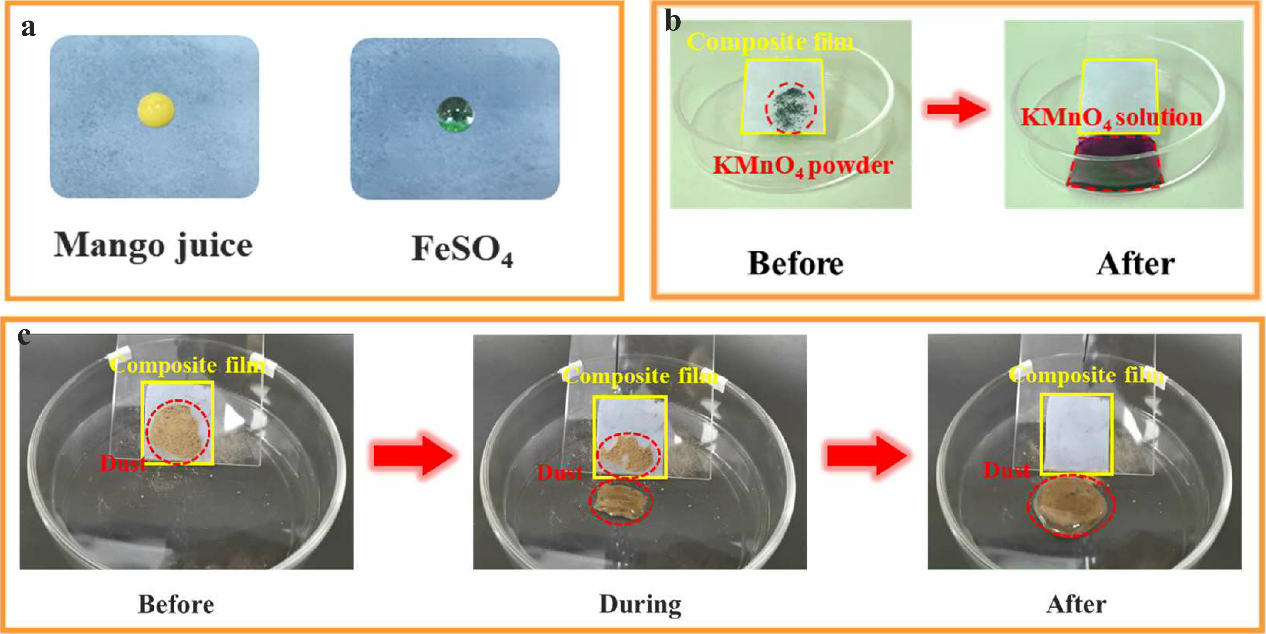
**

**Fig. S5** Self-cleaning performance of the composite film. **a** Hydrophobic droplet morphology of mango juice and FeSO_4_ on the composite film. **b** Self-cleaning performance of the composite film coated with KMnO_4_ powder. **c** Outdoor extreme contamination condition with 2 mm thick dust spread on the surface of composite film to verify the self-cleaning property at extreme contamination

**
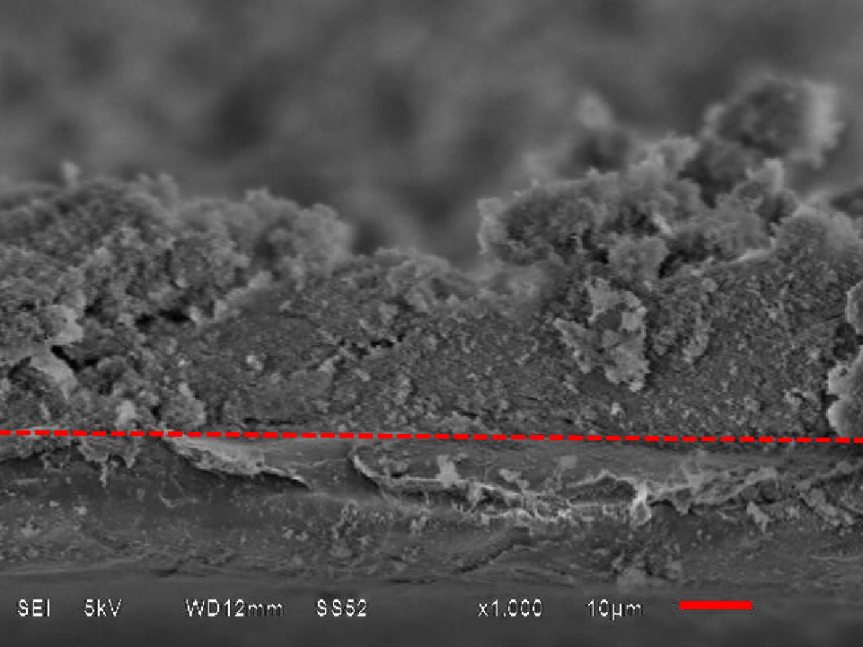
**

**Fig. S6** x1000 magnification of the cross-section of a PEAB-Si sample. The thickness of the coating is estimated to be 25 μm

**
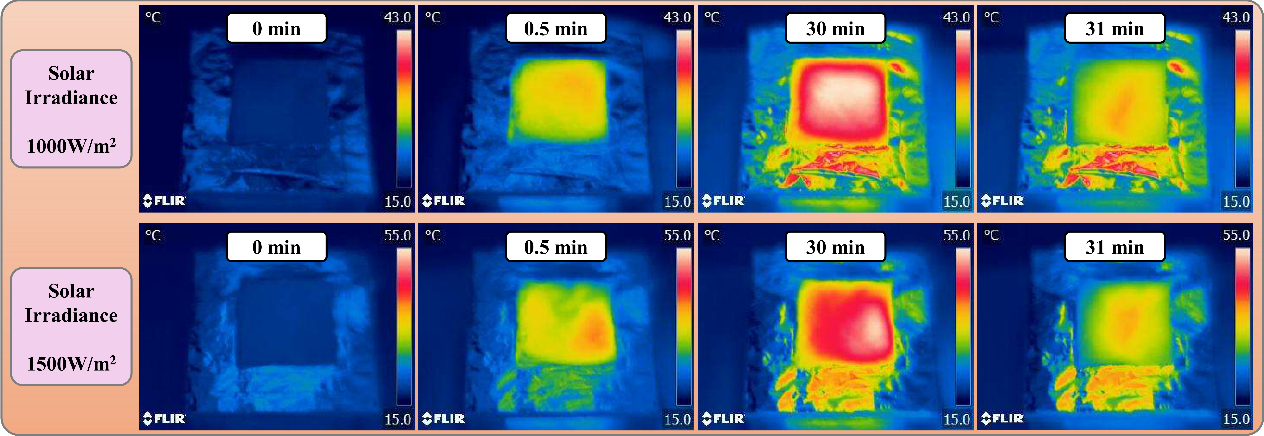
**

**Fig. S7** Infrared images of a cavity covered with a composite film after 30 minutes of direct illumination at light intensities of 1000 W/m^2^ and 1500 W/m^2^ using a sunlight simulator.

**
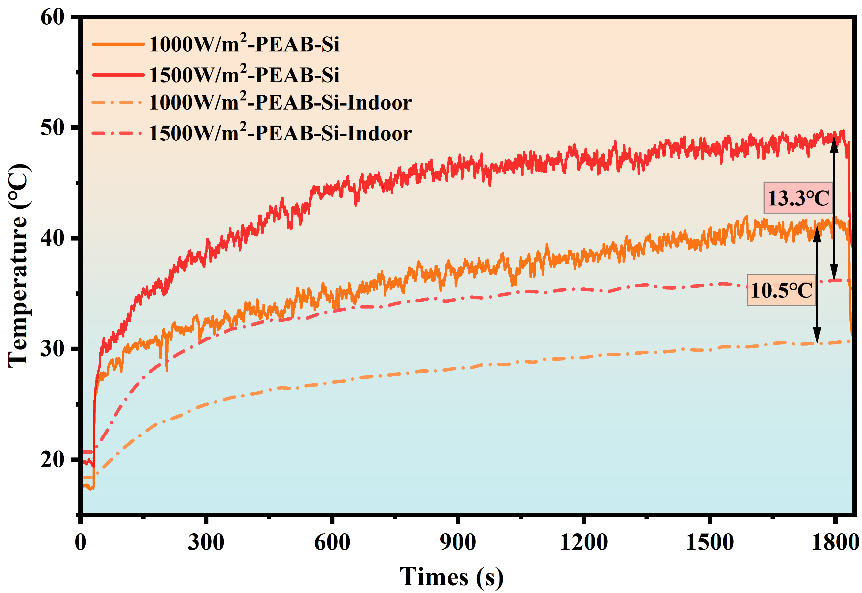
**

**Fig. S8** The temperature trends of the composite film's surface and the cavity interior under direct irradiation at varying light intensities were analyzed. The data reveal that, after 10 minutes of irradiation under 1000 and 1500 W/m^2^ illumination, the temperature differences between the film's surface and the cavity interior stabilize at approximately 10 and 13 ℃, respectively. Even after 30 minutes, these temperature differences remain stable at around 10.5 and 13.3 ℃

**
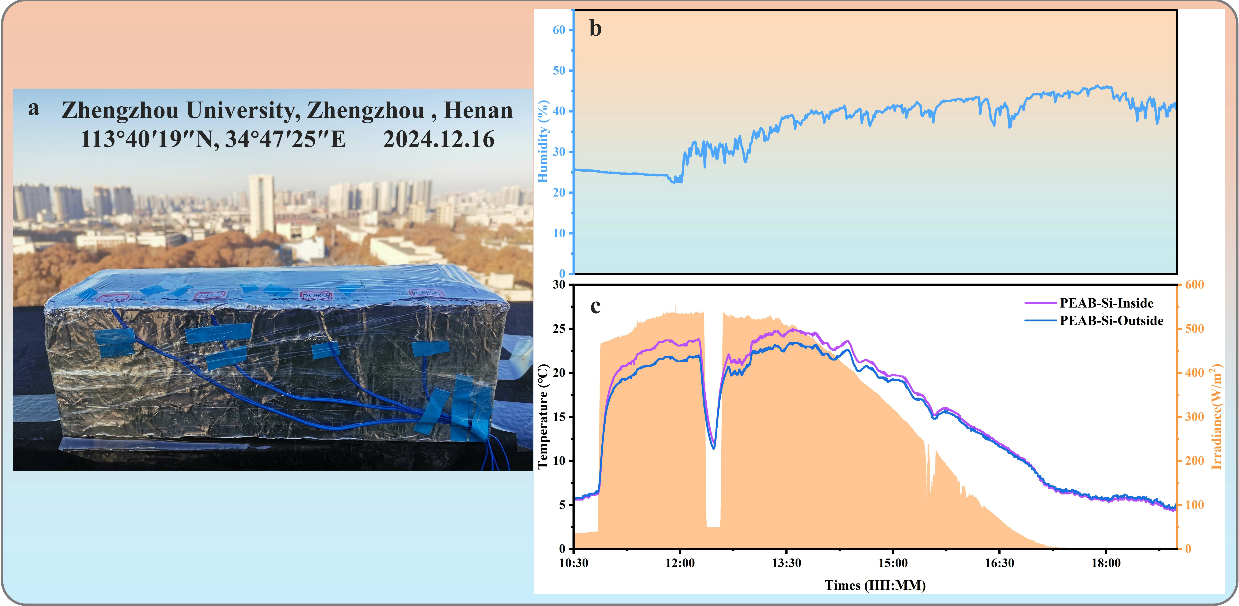
**

**Fig. S9** Outdoor tests were conducted under sunny winter conditions. **a** Photograph of the test setup. **b** humidity data recorded during the test. **c** internal temperature variations and light intensity for PEAB-Si films placed on the outside and inside of the chamber. During the daytime, the cavity temperature of the samples placed on the outside is, on average, 1-2 ℃ lower than that of the samples placed on the inside

**
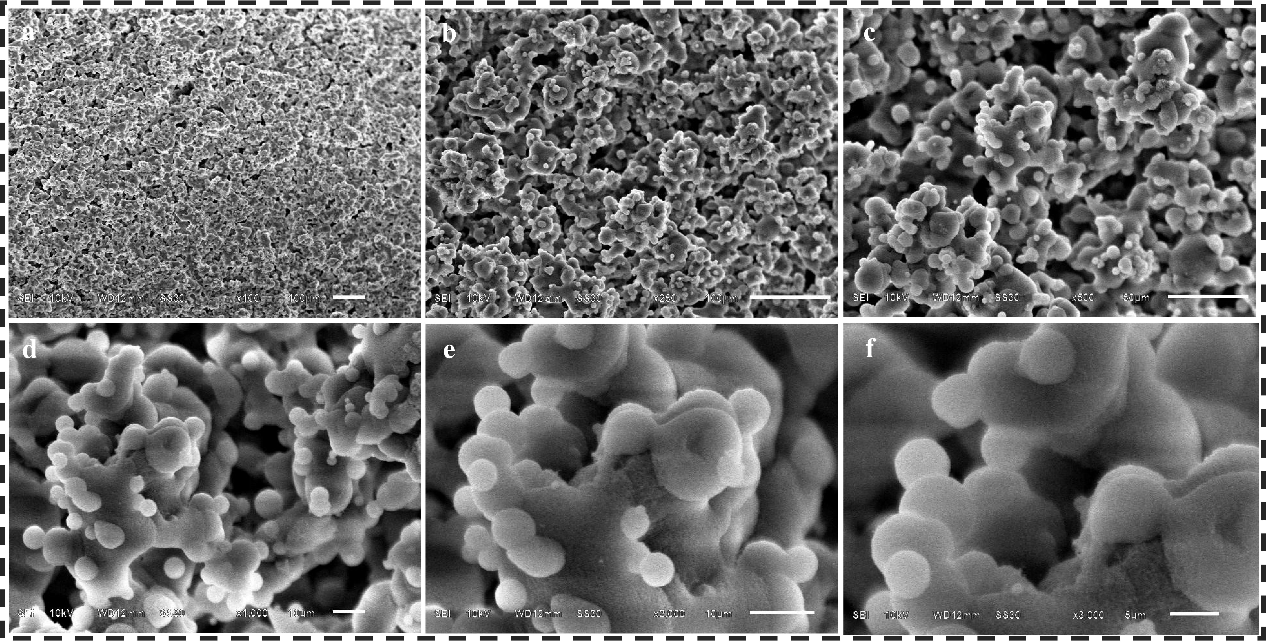
**

**Fig. S10** Micro-nanostructures of the PEAB-Si composite film surface. **a-f** SEM images of the surface morphology at magnifications of ×100, ×250, ×500, ×1000, ×2000, and ×3000, respectively

**
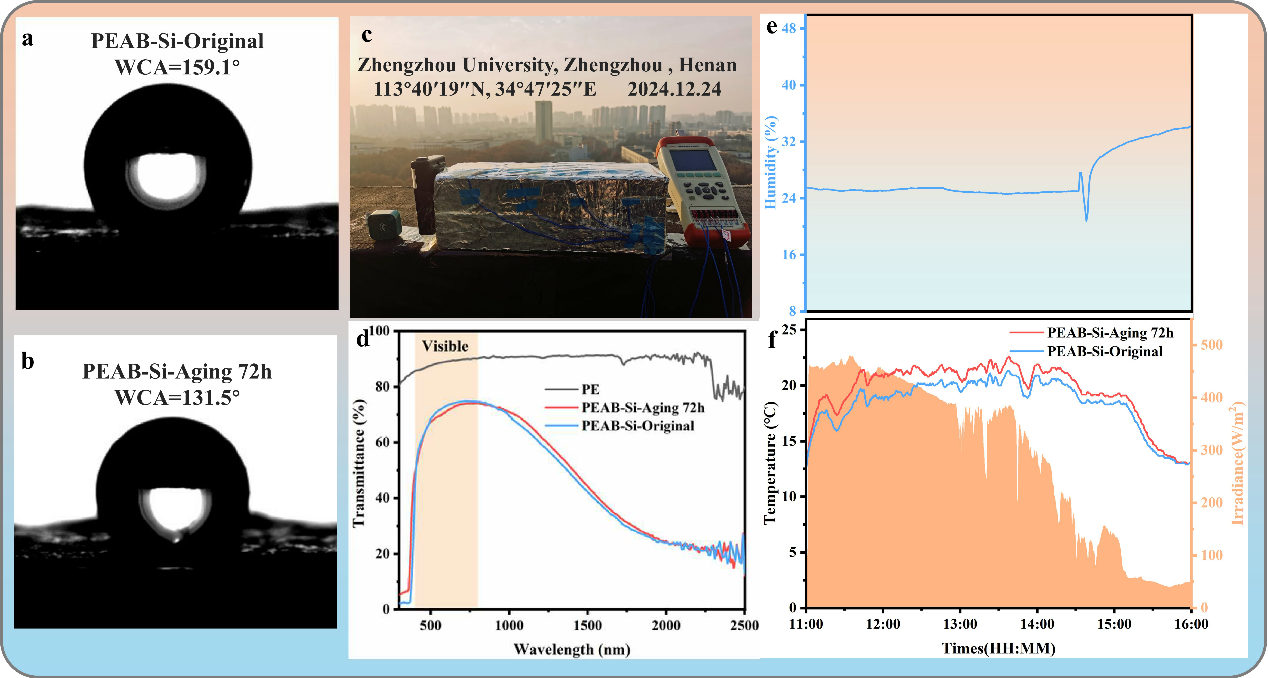
**

**Fig. S11** Properties of PEAB-Si samples aged at 90 ℃ for 72 hours. **a** Water contact angle of PEAB-Si samples before high-temperature aging: 159.1°. **b** Water contact angle of PEAB-Si samples after high-temperature aging: 131.5°. **c** Outdoor testing of samples before and after high-temperature aging under natural winter light conditions. **d** Transmittance of samples before and after high-temperature aging across the solar spectrum (250–2500 nm). **e** Humidity data recorded on the day of the experiment. **f** Actual cooling performance and real-time light intensity of samples before and after high-temperature aging, the average temperature of the aged samples was 1-2 ℃ higher than that of the original samples

**
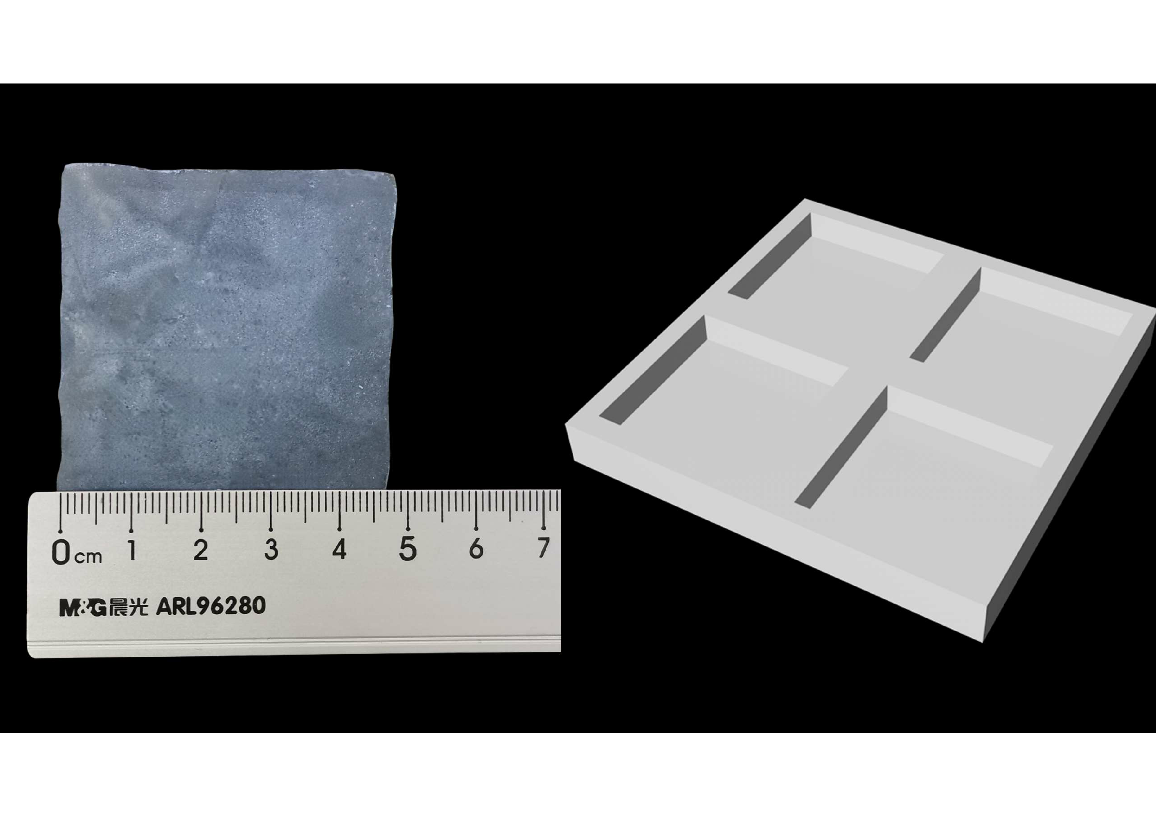
**

**Fig. S12** Actual scale of prepared samples and processing mold dimensions

**
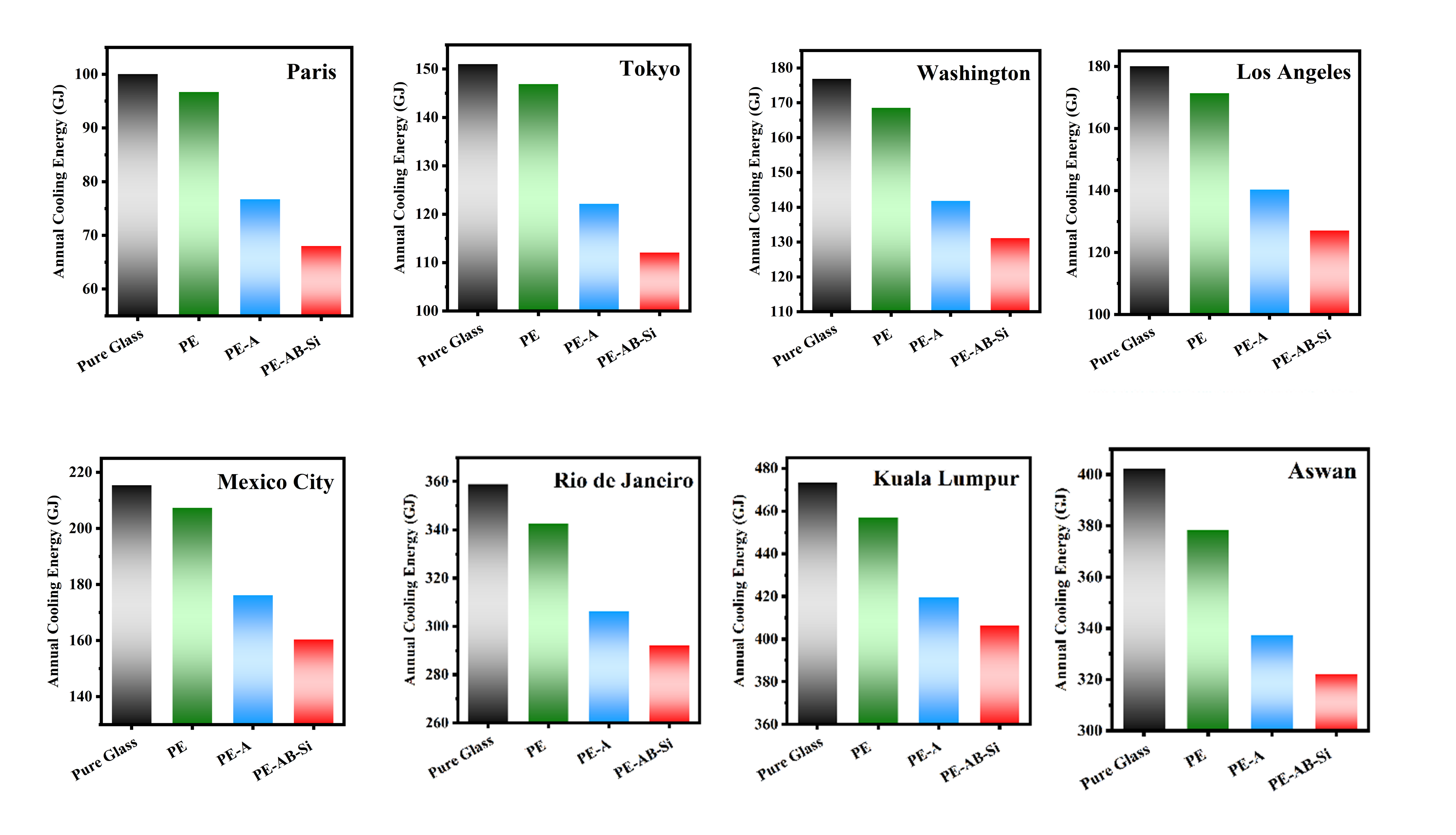
**

**Fig. S13** The annual cooling energy consumption of building models using these four types of windows based on weather data from 8 cities


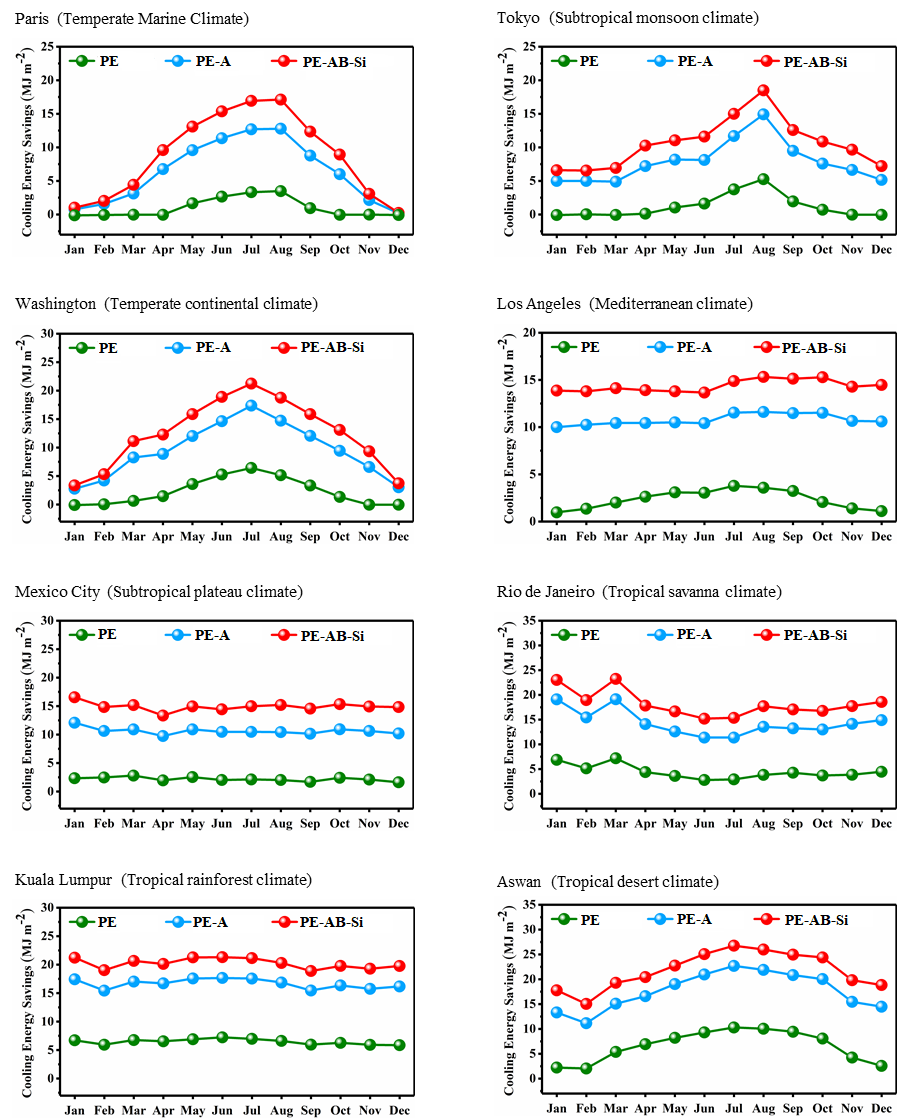


**Fig. S14** Extra cooling energy savings of building models using three types of windows from 8 cities each month


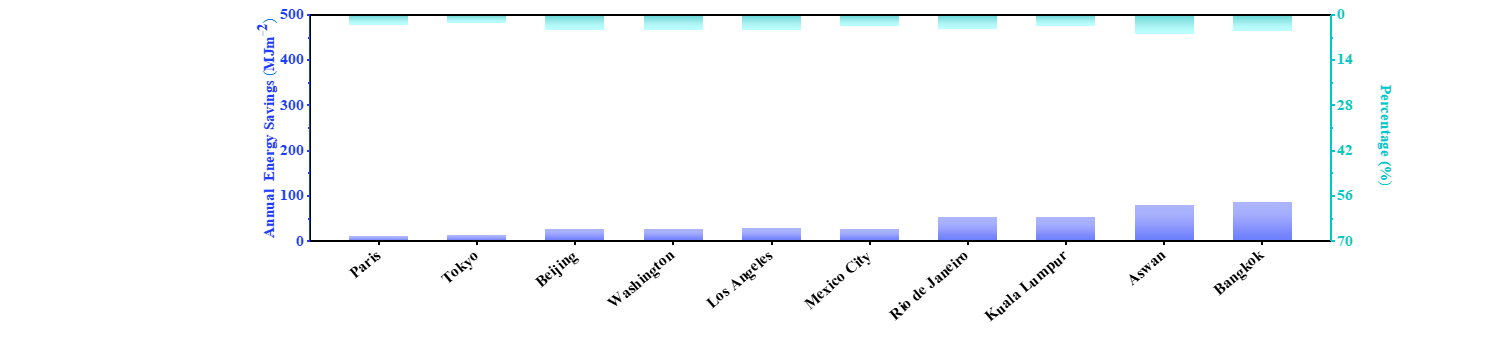


**Fig. S15** The annual cooling energy savings and percentage of building models using PE film based on weather data from 10 cities


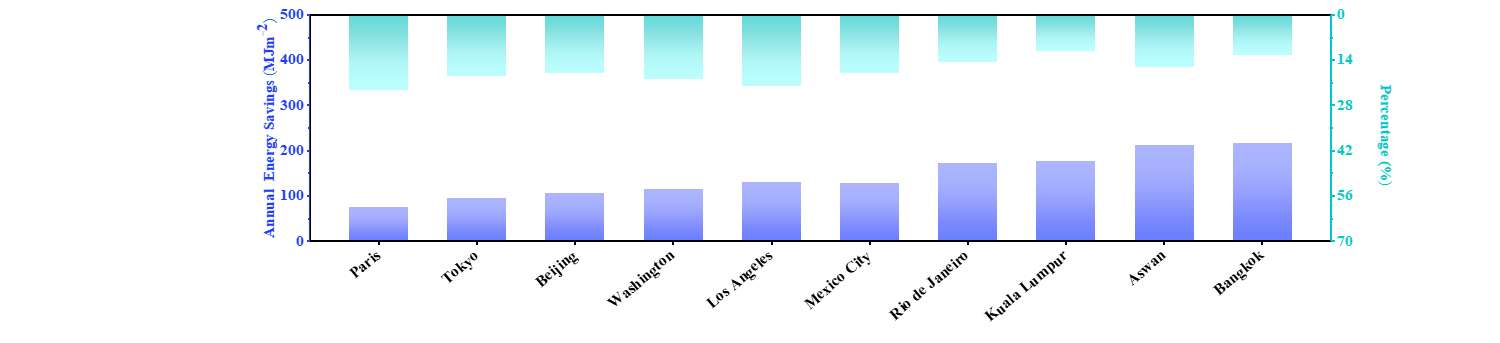


**Fig. S16** The annual cooling energy savings and percentage of building models using PE-A film based on weather data from 10 cities

**Video S1** The dust (KMnO_4_ powder) was removed from the film surface

**Video S2** The water droplets remained intact and were removed by the needle

**Video S3** Excellent self-cleaning ability under extreme outdoor dust contamination conditions

**Table S1** The transmittance and haze of the composite film are compared with other polyethylene-based composite materials

| Composites | Transmission (%) | UV shielding (%) | NIR shielding (%) | Years | Refs. |
| --- | --- | --- | --- | --- | --- |
| VO_2_-NANO | 58 | ~50 | 70 | 2021 | [S2] |
| HEC/ANFs | 70 | ~80 | / | 2021 | [S3] |
| CA/Cu/MOF | 82 | ~60 | / | 2021 | [S4] |
| PVA/IC | 76 | ~80 | / | 2020 | [S5] |
| PVA/MnO_2_ | 12 | 92 | / | 2018 | [S6] |
| GO/PVA | 30 | ~88 | / | 2015 | [S7] |
| PVA/CuS | 56 | ~55 | / | 2020 | [S8] |
| TiO_2_/PDMS | 92 | ~28 | / | 2017 | [S9] |
| PVA/LS | ~80 | ~32 | / | 2021 | [S10] |
| PEI/CMC@TiO_2_ | ~42 | ~75 | / | 2017 | [S11] |
| MUVS | ~56 | ~95 | / | 2020 | [S12] |
| GO/Cellulose | ~72 | ~45 | / | 2020 | [S13] |
| CNF/PVA/ATO-10 | ~90 | / | ~9 | 2021 | [S14] |
| CNF/PVA/ATO-40 | ~83 | / | ~47 | 2021 | [S14] |
| CNF/PVA/ATO-0 | ~49 | / | ~95 | 2021 | [S14] |
| ITO/Graphene | ~49 | / | ~45 | 2015 | [S15] |
| WO_2.72_/PU | ~55 | / | ~90 | 2017 | [S16] |
| W_17_O_47_ | ~56 | / | ~72 | 2021 | [S17] |
| PVC/ATO | 44 | / | 57 | 2016 | [S18] |
| AZO/Epoxy | 50 | / | 63 | 2011 | [S19] |
| LaB_6_@SiO_2_-PVB | 28 | / | 84 | 2014 | [S20] |
| AAA/CuS | 63 | / | 76 | 2017 | [S21] |
| Na_x_WO_3_ | 57 | / | 77 | 2012 | [S22] |
| This work | 77 | 91 | 74 | This work | |

The HEC/ANFs: hydroxyethyl cellulose/aramid nanofibers; CA/Cu/MOF: cellulose acetate/flower-like metal-organic frameworks; PVA/IC: poly(vinyl alcohol)/instant coffee; GO: Graphene oxide; PDMS: polydimethylsiloxane; LS: sodium lignosulfonate; PEI/CMC: poly(ethylene imine)/carboxymethyl cellulose sodium; MUVS: multilayered artificial UV shielding skin; CNF/PVA/ATO: cellulose nanofiber/polyvinyl alcohol/antimony tin oxide; ITO: indium tin oxide; PU: polyurethane; ATO: antimony tin oxide; AZO: Al doped ZnO; PVB: Poly(vinyl butyral); AAA: acrylic-amino-alkyd baking varnish.

**Table S2** Geographical coordinates and climate types of 10 cities

| City | Geographical coordinates | Climatic zones | Climatic types |
| --- | --- | --- | --- |
| Beijing (China) | 39º48′N, 116º28′E | Temperate zone | Monsoon climate of medium latitudes |
| Paris (France) | 48º51′N, 2º2′E |  | Temperate marine climate |
| Washington (USA) | 38º53′N, 77º02′W |  | Temperate continental climate |
| Tokyo (Japan) | 35º41′N, 139º44′E | Subtropical zone | Subtropical monsoon climate |
| Mexico City (Mexico) | 19º25′N, 99º4′W |  | Subtropical plateau climate |
| Los Angeles (USA) | 34º05′N, 118º55′W |  | Mediterranean climate |
| Kuala Lumpur (Malaysia) | 3º08′N, 101º42′E | Tropical zone | Tropical rainforest climate |
| Bangkok (Thailand) | 13º5′N, 100º29′E |  | Tropical monsoon climate |
| Rio de Janeiro (Brazil) | 22º54′S, 43º15′W | Tropical zone | Tropical savanna climate |
| Aswan (Egypt) | 23º58′N, 32º52′E |  | Tropical desert climate |

**Supplementary References**

1. Weather Data, <https://www.energyplus.net/weather.>
2. S. Lee, S. Lee, H. Kang, S. Nahm, B. Kim et al., Flexible electrochromic and thermochromic hybrid smart window based on a highly durable ITO/graphene transparent electrode. Chem. Eng. J. **416**, 129028 (2021). <https://doi.org/10.1016/j.cej.2021.129028>
3. J. Huang, Z. Lu, J. Li, D. Ning, Z. Jin et al., Improved mechanical and ultraviolet shielding performances of hydroxyethyl cellulose film by using aramid nanofibers as additives. Carbohydr. Polym. **255**, 117330 (2021). <https://doi.org/10.1016/j.carbpol.2020.117330>
4. K. Chen, J. Yu, J. Huang, Q. Tang, H. Li et al., Improved mechanical, water vapor barrier and UV-shielding properties of cellulose acetate films with flower-like metal-organic framework nanoparticles. Int. J. Biol. Macromol. **167**, 1-9 (2021). <https://doi.org/10.1016/j.ijbiomac.2020.11.164>
5. Y. Lyu, X. Gu, Y. Mao, Green Composite of Instant Coffee and Poly(vinyl alcohol): An Excellent Transparent UV-Shielding Material with Superior Thermal-Oxidative Stability. Ind. Eng. Chem. Res. **59**, 8640-8648 (2020). <https://doi.org/10.1021/acs.iecr.0c00413>
6. H. Roy, M. Mollah, M. Islam, M. Susan, Poly(vinyl alcohol)-MnO_2_ nanocomposite films as UV-shielding materials. Polym. Bull. **75**, 5629-5643 (2018). <https://doi.org/10.1007/s00289-018-2355-5>
7. S. Xie, J. Zhao, B. Zhang, Z. Wang, H. Ma et al., Graphene Oxide Transparent Hybrid Film and Its Ultraviolet Shielding Property. ACS Appl. Mater. Interfaces **7**, 17558-17564 (2015). <https://doi.org/10.1021/acsami.5b04231>
8. H. Yuan, T. Li, Y. Wang, P. Ma, M. Du et al., Photoprotective and multifunctional polymer film with excellent near-infrared and UV shielding properties. Compos. Commun. **22**, 100443 (2020). <https://doi.org/10.1016/j.coco.2020.100443>
9. S. Jang, S. Kang, M. Choi, Multifunctional Moth-Eye TiO_2_/PDMS Pads with High Transmittance and UV Filtering. ACS Appl. Mater. Interfaces **9**, 44038-44044 (2017). <https://doi.org/10.1021/acsami.7b1550>
10. J. Cui, T. Lu, F. Li, Y. Wang, J. Lei et al., Flexible and transparent composite nanofibre membrane that was fabricated via a "green" electrospinning method for efficient particulate matter 2.5 capture. J. Colloid Interface Sci. **582**, 506-514 (2021). <https://doi.org/10.1016/j.jcis.2020.08.075>
11. X. Li, J. Lv, D. Li, L. Wang, Rapid fabrication of TiO_2_@carboxymethyl cellulose coatings capable of shielding UV, antifog and delaying support aging. Carbohydr. Polym. **169**, 398-405 (2017). <https://doi.org/10.1016/j.carbpol.2017.04.04>
12. F. Liang, Y. Fan, S. Kuang, S. Wang, Y. Wang et al., Layer-by-Layer Assembly of Nanofiber/Nanoparticle Artificial Skin for Strain-Insensitive UV Shielding and Visualized UV Detection. Adv. Mater. Technol. **5**, 1900976 (2020). <https://doi.org/10.1002/admt.201900976>
13. X. Zhang, L. Song, Z. Wang, Y. Wang, L. Wan et al., Highly transparent graphene oxide/cellulose composite film bearing ultraviolet shielding property. Int. J. Biol. Macromol. **145**, 663-667 (2020). <https://doi.org/10.1016/j.ijbiomac.2019.12.241>
14. D. Fang, H. Yu, M. Dirican, Y. Tian, J. Xie et al., Disintegrable, transparent and mechanically robust high-performance antimony tin oxide/nanocellulose/polyvinyl alcohol thermal insulation films. Carbohydr. Polym. **266**, 118175 (2021). <https://doi.org/10.1016/j.carbpol.2021.118175>
15. S. Das, A. Salandrino, J. Wu, R. Hui, Near-infrared electro-optic modulator based on plasmonic graphene. Opt. Lett. **40**, 1516-1519 (2015). <https://doi.org/10.1364/OL.40.001516>
16. T. Chala, C. Wu, M. Chou, M. Gebeyehu, K. Cheng, Highly Efficient Near Infrared Photothermal Conversion Properties of Reduced Tungsten Oxide/Polyurethane Nanocomposites. Nanomaterials **7**, 191 (2017). <https://doi.org/10.3390/nano7070191>
17. J. Yan, A. Rath, H. Wang, Z. Ng, S. Pennycook et al., Tungsten Suboxide Nanoneedles as an Effective Thermal Shield through Near-Infrared Reflection and Absorption. J. Phys. Chem. C **125**, 11115-11123 (2021). <https://doi.org/10.1021/acs.jpcc.1c00449>
18. Y. Qi, X. Yin, J. Zhang, Transparent and heat-insulation plasticized polyvinyl chloride (PVC) thin film with solar spectrally selective property. Sol. Energy Mater. Sol. Cells **151**, 30-35 (2016). <https://doi.org/10.1016/j.solmat.2016.02.016>
19. Y. Li, Y. Kang, H. Xiao, S. Mei, G. Zhang et al., Preparation and characterization of transparent Al doped ZnO/epoxy composite as thermal-insulating coating. Compos. Pt. B-Eng. **42**, 2176-2180 (2011). <https://doi.org/10.1016/j.compositesb.2011.05.015>
20. H. Tang, Y. Su, J. Tan, T. Hu, J. Gong et al., Optical properties and thermal stability of poly(vinyl butyral) films embedded with LaB_6_@SiO_2_ core-shell nanoparticles. Superlattices Microstruct. **75**, 908-915 (2014). <https://doi.org/10.1016/j.spmi.2014.09.020>
21. L. Han, H. Li, Y. Lei, D. Cao, Preparation and transparent heat insulating properties of aqueous acrylic-amino-alkyd coatings with CuS nanoplates. Journal of Materials Science: Materials in Electronics **28**, 14596-14604 (2017). <https://doi.org/10.1007/s10854-017-7324-5>
22. C. Guo, S. Yin, T. Sato, Effects of Crystallization Atmospheres on the Near-Infrared Absorbtion and Electroconductive Properties of Tungsten Bronze Type M_x_WO_3_ (M = Na, K). J. Am. Ceram. Soc. **95**, 1634-1639 (2012). <https://doi.org/10.1111/j.1551-2916.2011.05039.x>
